# Eumycetoma caused by *Cladophialophora bantiana* in the United States

**DOI:** 10.1099/acmi.0.000030

**Published:** 2019-06-06

**Authors:** Thomas J. Gniadek, Mark A. Cappel, Nancy L. Wengenack, Claudia R. Libertin

**Affiliations:** 1 Division of Clinical Microbiology, Department of Laboratory Medicine and Pathology, Mayo Clinic, Rochester, MN 55905, USA; 2 Department of Dermatology, Mayo Clinic, Jacksonville, FL 32224, USA; 3 Division of Infectious Disease, Department of Medicine, Mayo Clinic, Jacksonville, FL 32224, USA; ^†^​ Present address: NortShore University Health System, Evanston, IL 60201, USA.; ^‡^​ Present address: Gult Coast Dermatopathology Laboratory, 6001 Memorial Highway, Tampa, FL 33615, USA.

**Keywords:** *Cladophialophora bantiana*, Eumycetoma, antifungal medication

## Abstract

A female presented in the sixth decade of life with a history of undifferentiated small cell carcinoma of the right breast in clinical remission, status-post chemotherapy and resection 6 years previously, presented with a chronic anterior knee skin nodule that grew in size over the prior 5–6 weeks. She had no history of opportunistic infections or recent immunosuppression. As it grew, the nodule became tender to touch. Examination revealed a 4–6 mm superficial purple-red nodule. Also, a similar lesion was present on the dorsum of her left foot for the past year, which also recently grew and became tender. The patient did report frequently kneeling on soil when gardening in Florida. She reported no other symptoms. Due to a concern for cutaneous metastasis of the patient’s previously diagnosed small cell carcinoma of the breast, the anterior knee lesion was biopsied. Histology revealed histocyte-rich inflammation with foci of acute inflammation as well as pigmented fungal forms. Subsequent fungal culture of excised tissue grew *Cladophialophora bantiana*, identified by ribosomal gene DNA sequencing.

## Introduction

Localized cutaneous fungal infections caused by soil-dwelling, dematiaceous fungi are traditionally associated with exposures in tropical and developing countries. However, these infections can also occur in the developed areas, but their clinical presentation may not be immediately recognized. *Cladophialophora bantiana* is known to cause potentially fatal central nervous system (CNS) disease even in immunocompetent and otherwise healthy individuals [1]. Since culture isolates are an infectious risk due to inhalation of spores, biosafety level 3 precautions are typically practiced by laboratory workers [2]. However, this organism is widespread in the environment, especially in warm moist climates, including in the United States where it can be acquired through exposure to soil [3]. Even if the infection is peripheral and localized, awareness of the potential for CNS disease is important when evaluating any patient infected with this organism and ensuring the safety of laboratory staff [4].

## Case report

### Investigations

**Fig. 1.**
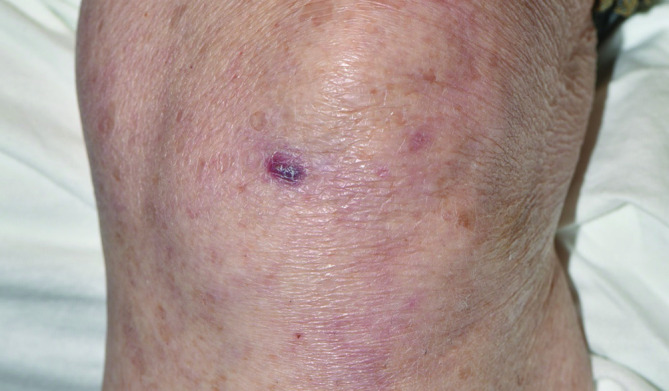
Nodule on the anterior aspect of the left knee (arrow). The nodule appeared dark red to purple, with a white scaly surface.

**Fig. 2.**
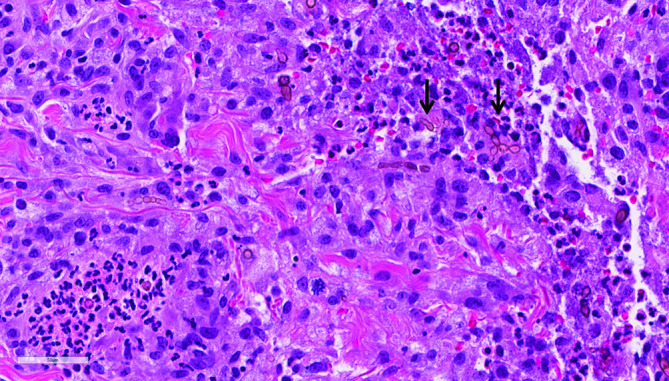
Haematoxylin and eosin stain of left knee lesion biopsy showing pigmented fungal elements, including short hyphae and chains of conidia (see arrows). Background tissue shows focal acute inflammation and histiocyte-rich chronic inflammation.

Punch biopsy of the left knee (Fig. 1) lesion demonstrated darkly pigmented fungal forms in a background of suppurative granulomatous inflammation within the dermis (Fig. 2), without overlying pseudoepitheliomatous hyperplasia. Gomori Methenamine-Silver Nitrate Stain (GMS) highlighted the fungal elements, while an acid fast bacillus (AFB) stain was negative. Fungal cultures were performed.

Fungal culture from the punch biopsy was positive with mold growth on Inhibitory Mold Agar after 4 days. Mold colonies were black on both the top and reverse sides. Microscopic morphology using a lactophenol cotton blue stain demonstrated long, wavy chains of oval conidia. Sequencing of a 300 base pair D2 region of the 28S ribosomal RNA gene gave a 100 % match to *C. bantiana* using a custom, in-house DNA library [5].

Excisional biopsy, performed 1.5 months after the punch biopsy, showed a dermal scar and giant cell reaction, but no acute inflammation or fungal organisms (GMS stain was negative). MRI revealed no visible CNS disease.

### Diagnosis

Diagnosis of eumycotic mycetoma was made based on the culture results and clinical findings.

### Treatment

The patient was treated with wide local excision of the lesions, followed by treatment with voriconazole for 3 months. Healthcare workers wore surgical facemasks and gloves as a precaution when examining the patient and performing the excisional biopsy.

### Outcome and follow up

The patient completed voriconazole therapy. The patient’s post-excisional wounds healed well, with no evidence of recurrence, and the patient had no other symptoms of disease. There were no reports of fungal infection or unexplained CNS disease among healthcare workers, laboratory staff, or the patient’s close contacts.

## Discussion

The literature reports multiple cases of fatal CNS disease due to *C. bantiana* [1]. Affected patients can be otherwise immunocompetent and healthy, unlike many environmental organisms that produce disease most often in immunocompromised individuals. Infections can occur through direct contact with soil (inoculation) or inhalation of conidial spores [3, 6].

This case illustrates that *C. bantiana* can also present as an indolent localized cutaneous infection (mycetoma) even in patients without a remarkable travel history. This is important to consider due to the potential for CNS involvement and the potential hazard to clinical and laboratory staff. Patients with this infection should likely be screened by imaging studies of the CNS to rule out an abscess and the clinical and laboratory staff should be cautioned to take exposure precautions with any biopsy or cultured materials [4, 7].

The presence of conidia on tissue biopsy raises the question of whether skin infections with *C. bantiana* can lead to person-to-person transmission, particularly in the patient care setting. To our knowledge, no such transmission has been reported in the literature [8].
